# Cellular organization and molecular differentiation model of breast cancer-associated fibroblasts

**DOI:** 10.1186/s12943-017-0642-7

**Published:** 2017-04-03

**Authors:** Susann Busch, Daniel Andersson, Eva Bom, Claire Walsh, Anders Ståhlberg, Göran Landberg

**Affiliations:** 1 Department of Pathology, Sahlgrenska Cancer Center, Institute of Biomedicine, Sahlgrenska Academy, University of Gothenburg, Gothenburg, Sweden; 2 Department of Surgery, Institute of Clinical Sciences, Transplantation and Regenerative Medicine, Sahlgrenska Academy, University of Gothenburg, Gothenburg, Sweden

**Keywords:** Cancer-associated fibroblast, Breast cancer, Tumour stroma, Single-cell analysis

## Abstract

**Background:**

The role of cancer-associated fibroblasts (CAFs) during tumour progression is obscured by the inherently complex, heterotypic nature of fibroblast cells and behaviours in various subtypes of malignancies. Therefore, we sought to identify distinct fibroblast subpopulations at the single-cell level.

**Methods:**

Using single-cell quantitative PCR as a powerful tool to study heterogeneity and rare cell events, in a high-throughput manner a panel of gene targets are run simultaneously on transcripts isolated from single cells obtained by fluorescence-activated cell sort. Assessment of cells with stem-like characteristics was attained by anchorage-independent, anoikis-resistant culture.

**Results:**

Single-cell analysis of fibroblasts and their tumour-activated counterparts demonstrated molecularly distinct cell types defined by differential expression of characteristic mesenchymal and fibroblast activation markers. Identified subpopulations presented overlapping gene expression patterns indicating transitional molecular states during fibroblast differentiation. Using single-cell resolution data we generated a molecular differentiation model which enabled the classification of patient-derived fibroblasts, validating our modelling approach. Remarkably, a subset of fibroblasts displayed expression of pluripotency markers, which was enriched for in non-adherent conditions. Yet the ability to form single-cell derived spheres was generally reduced in CAFs and upon fibroblast activation through TGFβ1 ligand and cancer cell-secreted factors. Hence, our data imply the existence of putative stem/progenitor cells as a physiological feature of undifferentiated fibroblasts.

**Conclusions:**

Within this comprehensive study we have identified distinct and intersecting molecular profiles defining fibroblast activation states and propose that underlying cellular heterogeneity, fibroblasts are hierarchically organized. Understanding the molecular make-up of cellular organization and differentiation routes will facilitate the discovery of more specific markers for stromal subtypes and targets for anti-stromal therapies.

**Electronic supplementary material:**

The online version of this article (doi:10.1186/s12943-017-0642-7) contains supplementary material, which is available to authorized users.

## Background

With the need of more personalized medicine, targeting the tumour microenvironment/stroma has become an increasingly relevant and emerging concept. A permissive or tumour-promoting stroma coevolves with cancer cells during tumourigenesis [1] and in breast cancer, cancer-associated fibroblasts (CAFs) are the most prominent stromal cell type. CAFs thus present an attractive treatment option. Yet, the heterogenic nature and functional complexity of CAFs, although well-known, has been greatly understudied.

Physiologically, fibroblasts are mesenchymal cells and as the main cellular component of connective tissues maintain tissue homeostasis. Fibroblasts deposit and remodel extracellular matrix (ECM) and facilitate wound healing upon injury-induced activation. Transforming growth factor-beta (TGFβ) is the most potent inducer of fibroblast transformation into ‘activated’ fibroblasts with elevated smooth muscle actin-alpha (SMAα) levels (encoded by *ACTA2*). This myodifferentiation gives rise to a contractile and secretory cell, a phenotype which has been associated with CAFs [2, 3]. However, although genetically stable, CAFs display a vast cellular heterogeneity and thus far no unique marker or common predominant pathway has been identified. Classically CAFs are assigned with pro-tumorigenic qualities. Recent advances however have revealed tumour-inhibitory features [4] along with cancer subtype-specific characteristics [5]. This suggests that tumour-residing fibroblasts manifest not only phenotypic but also functional plasticity and therefore necessitating a more extensive understanding of the origin and biology of CAFs.

This study aims to dissect cellular composition constituting fibroblast heterogeneity on single-cell level enabling identification and characterization of molecular subsets of breast cancer-associated fibroblast subpopulations with reference to normal tissue-resident fibroblasts. Single-cell resolution data provided us with in-depth information for adequate modelling of fibroblast subsets. As a result, generation of a molecular fibroblast differentiation model enabled us to categorize patient-derived fibroblasts regarding its activation state. We therefore report herein proof-of-principle how to identify and characterize individual tumour stroma subtypes. We further provide first evidence of fibroblast stem or progenitor cells signifying a cellular hierarchy.

We have previously hypothesized that the existence of functional and phenotypic diverse fibroblast subpopulations may reflect either [a] different stages during fibroblast activation which may either be transitory or represent irreversible cell types, [b] different cells of origin (such as tissue-resident fibroblasts, recruitment of circulating mesenchymal stem cells or fibrocytes), [c] distinct modes of activation (eg. cancer subtype, chemokine profile or physical tension), [d] a stochastic/ hierarchical organization, or likely [e] a combination of thereof [6]. In line with this and the presented study, we propose that the observed heterogeneity of CAFs is a consequence of transitional molecular states with overlapping marker expression during fibroblast activation with an underlying hierarchical program.

## Methods

### Cell culture and fibroblast isolation

Control fibroblasts and expCAFs cells are a kind gift of Dr Akira Orimo. The generation of these cell lines have been described previously [7]. Fibroblast cell lines were cultured in DMEM with 10% foetal bovine serum (FBS) in a humid chamber with 5% CO_2_ at 37 °C. Primary CAFs were isolated from surgically resected invasive breast carcinomas on the day of surgery. Normal-matched control tissue was taken approximately 2 cm distant from tumour area. Tumour and normal tissue were mechanically dissected and subjected to explant outgrowth and cultured in DMEM supplemented with 20% FBS and 1% non-essential amino acids in a humid chamber at 37 °C with 5% CO_2_ and at a physiological level of 5% O_2_. Outgrown cells were trypsinized and filtered through a 100um cell strainer to obtain single-cell suspension for further propagation. Throughout cell culture patient-derived fibroblast cells sequentially outnumbered tumour cells which are sensitive to sequential passaging and epithelial cell clusters disappeared after passage three. Enriched fibroblast cells were cultured up to ten passages. Fibroblastic nature was assessed by microscopic assessment of characteristic spindle-like cell morphology (Additional file 1A). For clinical information of all utilized tumours refer to Additional file 1B.

### Single-cell gene expression profiling

Procedure has been described previously [8]. Briefly, single cells were obtained by fluorescence-activated cell sorting (FACS Aria II, BD Biosciences) into a 96-well plate excluding non-viable (7AAD+) cells and subjected to direct cell lysis in 5ul containing 1ug/ul BSA in DNase/RNase-free water, followed by immediate freezing on dry ice. RNA was reversed transcribed and sort efficiency was monitored by measuring *GAPDH*. Obtained cDNA was preamplified using a pool of gene-specific primers. Samples were prepared for high-throughput real-time quantitative PCR using Fluidigm platform to assess gene expression levels of selected genes. Obtained single-cell qPCR data was pre-processed and subjected to multivariate analysis using GenEx (Multid Analyses, Version 5) as has been described [9]. No normalization to reference gene was performed, instead gene expression data are presented per cell. For principle component analysis (PCA) data were autoscaled per gene and for unsupervised clustering (heatmaps) data were mean-centred by gene using log2-transformed data unless otherwise specified. To compensate for variations in absolute RNA expression levels, data in Fig. 2b were autoscaled by cell which corresponds to a global normalization and standardizes expression values to a common scale. Distribution of *ACTA2* gene expression levels were computed in Graphpad Prism (Version 5.01).

### Anoikis resistance and sphere formation assay

Adherent fibroblast cells were trypsinized, washed with PBS and syringed with 25-gauge needle to obtain a single-cell suspension. Triplicates of 5000 cells were seeded in 2 ml phenolred-free DMEM/F12 supplemented with 2% B27 serum (Gibco) into non-adherent, poly(2-HEMA)-coated 6-well plates. Number of spheres larger than 50um was assessed after 5 to 6 days. Cells were treated with either 10 ng/ml recombinant human TGFβ1 (rhTGFβ1), 10uM SB431542 (TGFBR1 inhibitor), 10uM LY2109761 (TGBFR1/TGFBR2 inhibitor), LY2157299 (TGFBR1 inhibitor) or 72 h cell-conditioned media of ERα-positive breast cancer cell line MCF7 or ERα-negative breast cancer cell line MDA-MB-231 for 48 h prior to sphere formation assay. Data are shown in mean+/-SEM and two-way analysis of variance (ANOVA) with replicates was performed for statistical analysis using Graphpad Prism (Version 5.01). For anoikis-resistance, single-cell suspension setup was scaled up 20x to obtain sufficient number of viable cells after 24 h culture for subsequent RNA analysis.

### Label-retention assay

For PKH26 staining of sphere-forming cells, adherent fibroblast cells were trypsinized, washed with serum-free media, suspended in Diluent C and labelled with 1uM PKH26 dye for 3 min according to manufacturer’s instruction (Sigma-Aldrich). Stained cells were washed three times, syringed to obtain single cells and counted to seed 500 cells for sphere formation under non-adherent conditions as described in section above.

### Standard quantitative RT-PCR

Following 24 h anoikis resistance cells were collected, directly lysed and subjected to RNA isolation (Qiagen). RNA was transcribed using Grandscript Reverse Transcriptase (TATAA) and 20 ng of resulting cDNA was used for real-time quantitative PCR (Applied Biosystems 7900) using Sybrgreen (Bioline) and 0.4uM of the same target-specific primers used for single-cell gene expression profiling.

## Results

**Fig. 1:**
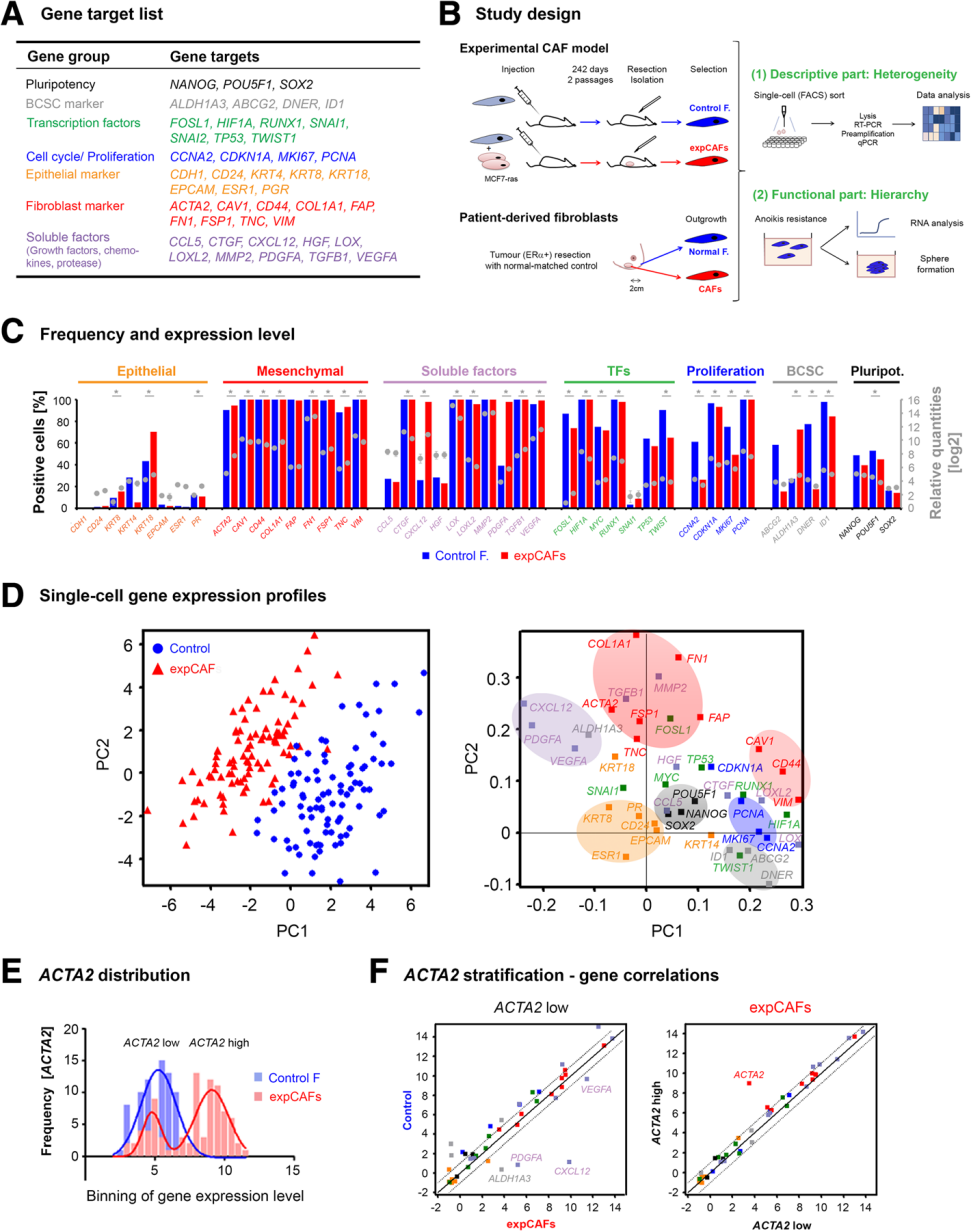
Molecular markers distinguish normal fibroblasts from the tumour-activated counterparts. **a** Table of selected gene targets colour-coded according to their attributes (gene group). **b** Schematic representation of study design. **c** Basic statistics of single-cell gene expression profiles of experimental fibroblast model. Graphs represent frequency of selected gene targets in percentage as bars and average gene expression levels given as log2-transformed relative quantities depicted as dots. Error bars represent SEM (*p-value < 0.05 Student *t*-test, Control F: control fibroblast, expCAF: experimentally-generated cancer-associated fibroblast). **d** (*Left panel*) Principal component analysis (PCA) of 183 individual Control fibroblasts (*n* = 92, *blue dots*) and expCAFs (*n* = 91, *red triangles*). In the principal component projections the position of a single cell (scores) is based on the expression of the analysed genes (*n* = 43), each dot represents a single cell. (*Right panel*) Plot represents the gene loadings for the PCA. Principal component projection of the genes illustrates the contribution of each gene to the scores of PC1 and PC2. Groups of genes are indicated as follows; *orange*: epithelial, *red*: fibroblast markers, *purple*: chemokines, *green*: transcription factors, *blue*: proliferation markers, *grey*: breast cancer-specific stem cell markers, *black*: pluripotency. **e** Histogram of distribution of *ACTA2*-specific gene expression with Gaussian regression analysis for control fibroblasts (*blue line*) and expCAFs (*red line*: sum of two Gaussians) demonstrating a bimodal gene expression pattern. **f** Plots depict correlations of gene expression between *ACTA2* low expressing expCAFs and Control fibroblasts (*left*) and *ACTA2* high expressing expCAFs (*right*). Genes outside the significance area (*dotted black line*) are denoted

To delineate cellular heterogeneity of fibroblasts we deployed a microfluidics platform (Fluidigm) for multiplex gene expression analysis of individual cells. This approach allows analysis of simultaneous gene expression and resolves cellular diversity at the single-cell level. We designed single-cell assays to study gene expression typically associated with mesenchymal cells and fibroblast activation alongside genes involved in pluripotency, breast cancer-specific stemness (BCSC), epithelial-to-mesenchymal transition, cell cycle and proliferation (see list of target genes in Fig. 1a). Single-cell assays and subsequent quantitative PCR procedure have previously been described by our group [8]. Briefly, we collected single cells using fluorescence-activated cell sorting excluding non-viable cells. Sort efficiency was monitored by measuring cells positive for *GAPDH* expression to verify >80% positivity. Samples were pre-amplified and subjected to high-throughput single-cell qPCR analysis using the Fluidigm platform. Workflow for processing of obtained single-cell data has been reported [9] and was applied accordingly. Only *GAPDH*-positive samples were included for downstream data analysis. For our study we employed an experimental CAF cell line model (expCAFs) [7] and patient-derived material for descriptive and functional analysis of normal fibroblasts and their breast cancer-activated counterparts (see schematic depiction of study design in Fig. 1b).

### Molecular markers characterizing fibroblast activation

Comparing frequencies of cells expressing relevant gene-specific targets demonstrates that fibroblast cells were positive for typical fibroblast markers (such as *VIM*, *CD44*) and mostly negative for epithelial markers (*CDH1, EPCAM*) (Fig. 1c). Characteristic fibroblast activation gene targets such as *ACTA2*, *COL1A1* and *TNC* were significantly upregulated in CAFs together with typical cancer-induced chemokines most prominently *CXCL12*, which has been reported for this cell line [7]. Concomitantly *CAV1* was downregulated in CAFs alongside other mesenchymal markers such as *CD44* and *VIM*. Loss of CAF-specific Caveolin-1 expression has been reported to be linked to worse prognosis in multiple cancers including breast cancer [10].

Based on the individual single-cell gene expression profiles, principle component analysis (PCA) revealed clearly distinguishable clusters of experimental CAFs when compared to their control counterparts (Fig. 1d). Strikingly, corresponding gene clusters were predominantly defined by gene groups (ie. pluripotency, BCSC-like, proliferation, fibroblast and epithelial markers) indicating potential co-regulation of a specific cellular function. However, expression patterns of transcription factors and soluble factors were more diverged, suggesting a more complex transcriptional regulation and induction of paracrine molecules.

SMAα is the most widely used marker to assess CAF activation status and is associated with worse clinical outcome [11]. Interestingly, we observed a bimodal distribution of expression level among the experimental CAF population (Fig. 1e), indicating a mixed population of *ACTA2* low and high expressing cells. Thus, in order to determine the role of SMAα in regard to fibroblast activation status we stratified cells according to their *ACTA2* expression level for correlation with other CAF activation markers. However, comparison of *ACTA2* low to *ACTA2* high expressing CAFs demonstrated no difference in gene expression profile other than *ACTA2.* Yet compared to control fibroblast, *ACTA2* low CAFs showed significant higher expression of *CXCL12, PDGFA, VEGFA* and *ALDH1A3* (Fig. 1f). These data suggest that CAFs with low SMAα expression do not correspond to a normal fibroblast phenotype but rather constitute a subset of activated CAFs with tumour-promotive features. Whether SMAα induction is in fact abrogated or merely bypassed using alternative fibroblast activation mechanisms needs to be clarified.

**Fig. 2:**
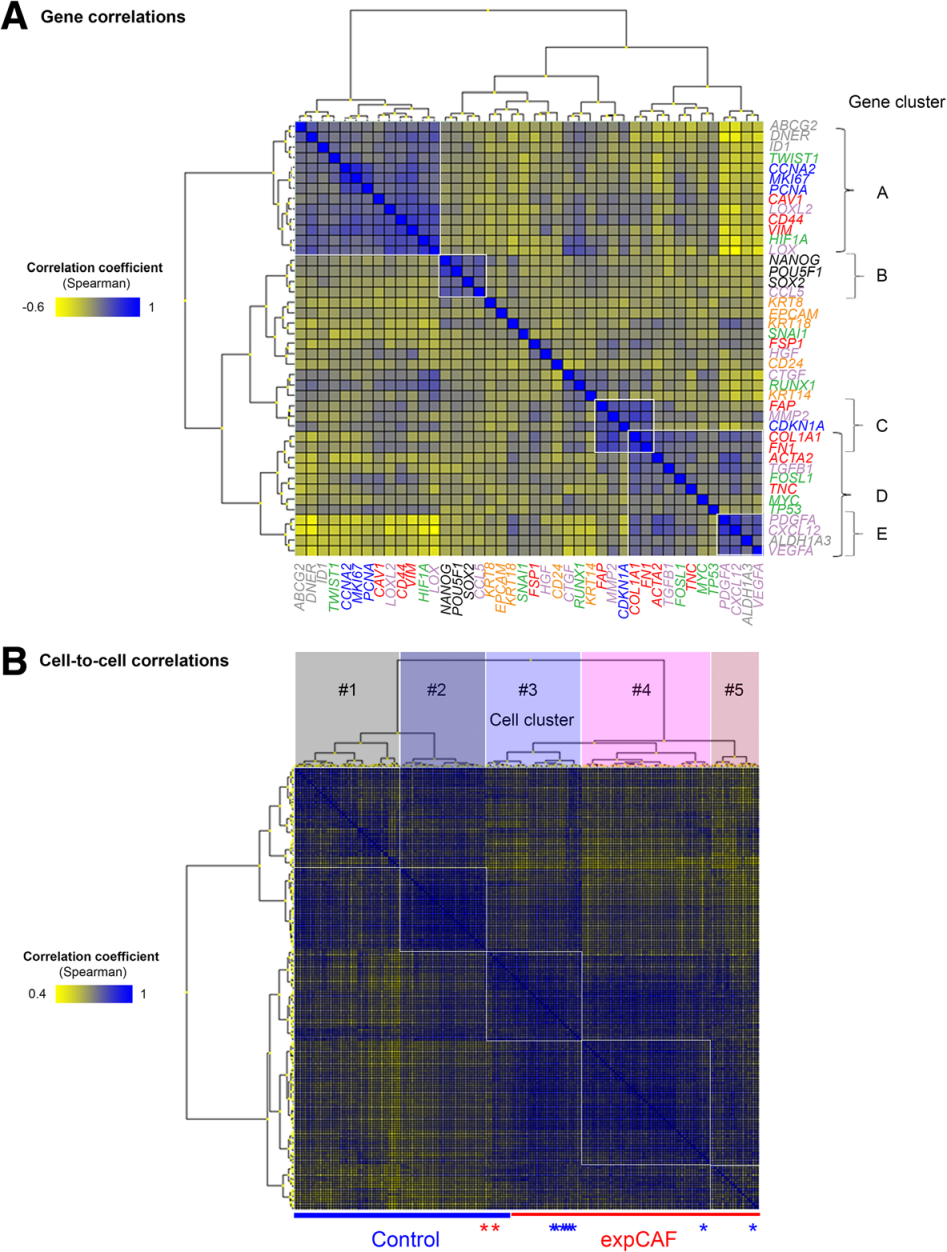
Gene and cell-to-cell correlation analysis. **a** Heatmap (unsupervised clustering, Euclidian distance) demonstrating gene correlations coefficients (Spearman) based on gene expression of all cells. **b** Heatmap (unsupervised clustering, Euclidian distance) demonstrating cell-to-cell correlations coefficients (Spearman) based on individual gene expression profile of each cell. Manual grouping was performed according to hierarchical clustering to obtain five subgroups

Gene correlation analysis confirmed observed PCA-projected gene clusters and highlighted five main clusters (Fig. 2a). Gene cluster A displays a strong correlation of *CAV1, CD44* and *VIM* with proliferation and BCSC markers. Strikingly, gene cluster B reveals correlation of all pluripotency markers with *CCL5*, which encodes for Rantes, a known mesenchymal stem cell effector [12] suggesting a distinct subpopulation with potential stem-like properties. The remaining clusters display overlap of correlation, fibroblast markers *FAP, COL1A1* and *FN1* (Cluster C) correlate with *ACTA2* and *TNC* (Cluster D) and to Cluster E including *PDGFA, CXCL12, VEGFA* and *ALDH1A3*. The latter interestingly shows a strong inverse correlation with gene cluster A. Remarkably when analysing gene correlation separately, correlating gene clusters including pluripotency were largely maintained within normal fibroblasts but either overlapping or absent in CAF cells (Additional file 2A, middle and left panel). This data indicates the presence of a stem-like subtype within normal fibroblasts and that CAFs may be more streamlined or in fact displaying transitional phenotypes rather than true (distinct) subtypes.

Collectively, our study confirms a distinct regulation of fibroblast-specific markers, including several indicators for aggressiveness, making them suitable markers for monitoring fibroblast activation. Whether downregulated expression of mesenchymal markers is a side-effect or a fundamental step of fibroblast activation needs to be further tested.

### Identification and modelling of subpopulations defining fibroblast differentiation states

**Fig. 3:**
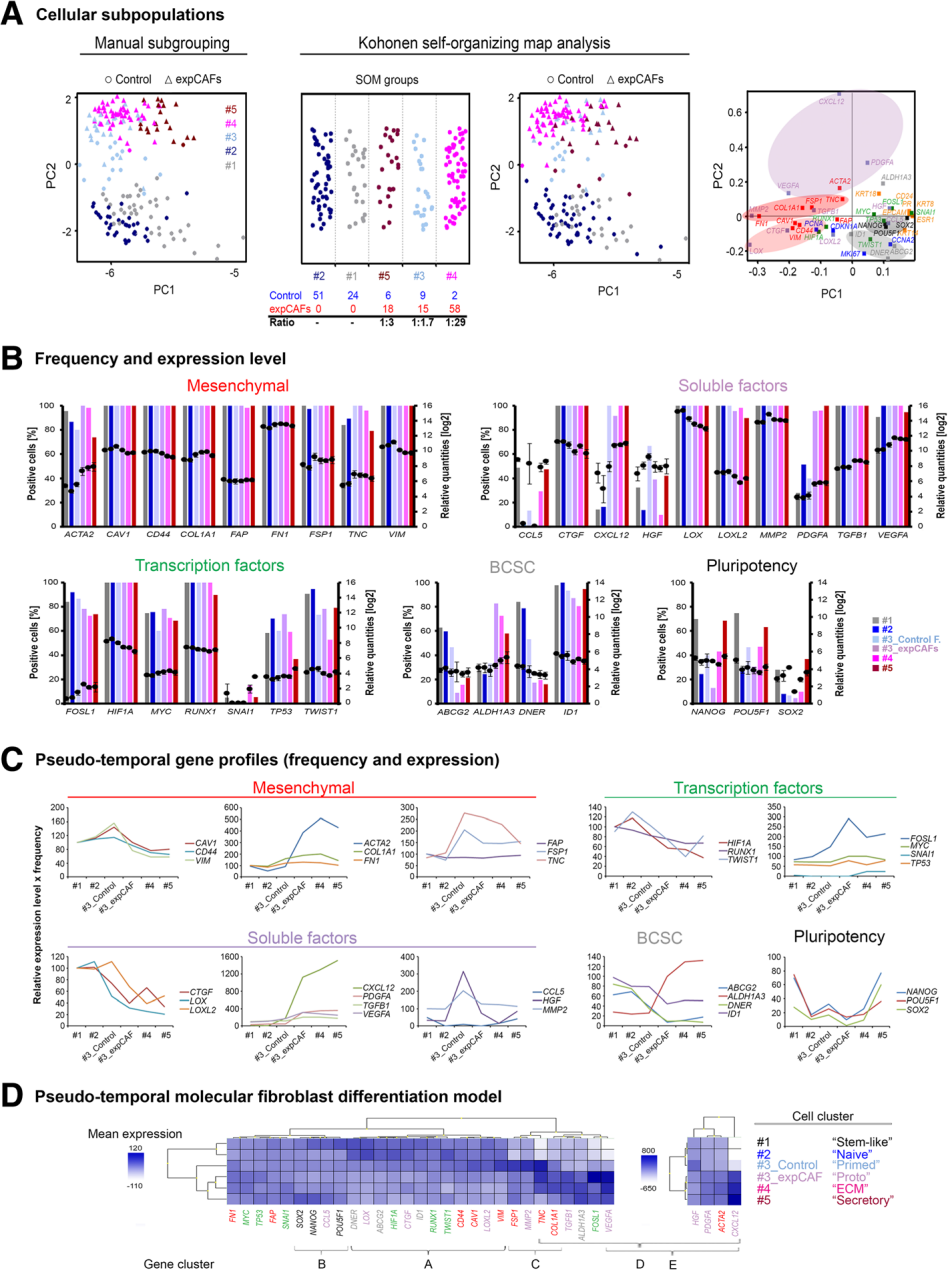
Identification of fibroblast subpopulations and generation of a molecular differentiation model. **a** Principal component analysis (PCA) of 183 individual normal (*n* = 92, dots) and experimentally generated cancer-associated fibroblasts (expCAFs, *n* = 91, triangles) colour-coded according to their subgroups. Data was normalized by autoscaling data per cell to eliminate differences due to global expression level (*left*). Kohonen self-organizing map (SOM) analysis confirms subgroups based on cell-to-cell correlation (*middle*). According gene loadings for PCA. Gene clusters are highlighted (*right*). **b** Basic statistics of single-cell gene expression profiles with regard to each subgroup. Bar graphs represent frequency of selected gene targets in percentage and average gene expression levels given as log2-transformed relative quantities are depicted as dots. Error bars represent SEM. **c** Graphs depict pseudo-temporal gene expression profile representing the product of relative expression level and frequency across subgroups. **d** Heatmap (unsupervised clustering) of pseudo-temporal gene profiles (combined frequency and relative average expression) per subgroup which were assigned a specific cellular state according to their most prominent gene transcripts thereby creating a pseudo-timeline of molecular differentiation states. Genes with extremely pronounced differences between subgroups were plotted separately for better visualization

To examine the existence of subpopulations at the single-cell level we performed cell-to-cell correlation analysis based on all analysed genes. The correlation coefficient (Spearman rho) which indicates how one cell relates to another is depicted in a heatmap, clustering cells by similarity (Fig. 2b). Cells were grouped according to hierarchical clustering of their correlation and consequently divided into five subgroups (Cell cluster #1-5) including two groups per cell type and one mixed subgroup (Cell cluster #3). Cell identification for clusters were noted and used for manual subgrouping of cells. In order to avoid skewing data by global RNA expression level, samples were normalized by autoscaling data per cell. As a result PCA analysis depicts clustering of identified cellular subgroups and also distinction of CAFs from normal counterparts (Fig. 3a). Strikingly, within cell cluster #3 normal fibroblasts show gradual conversion to more CAF-like phenotype. Similar cell clusters were observed when subgrouping samples applying self-organizing map (SOM) analysis. Projection of gene loadings for normalized cells was principally unaffected, displaying similar gene groupings (Fig. 3a).

For further analysis of frequency and average expression levels of gene targets per cell group, we divided mixed cell cluster #3 into control fibroblasts and expCAFs to assess potential differences in physiological fibroblast differentiation status and tumour-mediated fibroblast activation (Fig. 3b). We noticed that gene expression was mostly regulated by in- or decreased transcript level except for *HGF*, *CCL5,* pluripotency and BCSC-like target genes. Changes in number of positive cells may be indicative of a more switch-like gene regulatory mechanism of putative stem cells. To account for switch-like and transitional gene regulation equally, we generated pseudo-temporal gene expression profiles combining target-specific frequencies and relative expression levels for each cell cluster. Gene groups or similar gene expression profiles within the fibroblast and soluble factors gene transcripts are plotted together (Fig. 3c). According to their most prominent gene transcription we denoted normal fibroblast cell clusters as following; #1 as ‘stem-like’ (defined by high pluripotency expression), #2 ‘naive’ (lowest activation marker expression) and #3 as ‘primed’ (characterized with increased *MMP2, FSP1, TNC*, *COL1A1* and *FN1* expression) whereas expCAFs were denoted #4 as ‘proto-myofibroblasts’ (*ACTA2* expression is higher compared to control fibroblasts, but lowest amongst CAFs), #5 as ‘ECM-regulating myofibroblasts’ (highest *COL1A1* and *FN1* expression) and #6 as ‘secretory myofibroblasts’ (highest *CXCL12* and *PDGFA* expression). Based on selected markers unsupervised hierarchical clustering confirms much closer resemblance between ‘stem-like’ and ‘naive’ fibroblasts whereas ‘primed’ normal fibroblast were somewhat closer related to expCAFs, although still low in soluble factor secretion. According heatmap further illustrates proposed stepwise regulation of gene expression during fibroblast activation (Fig. 3d).

Overall, identifying distinct but overlapping gene expression patterns in fibroblast subpopulations allowed modelling of a transitional, progressive differentiation process and the regulation of pluripotency markers indicates an underlying hierarchical program.

### Molecular classification of patient-derived fibroblasts

**Fig. 4:**
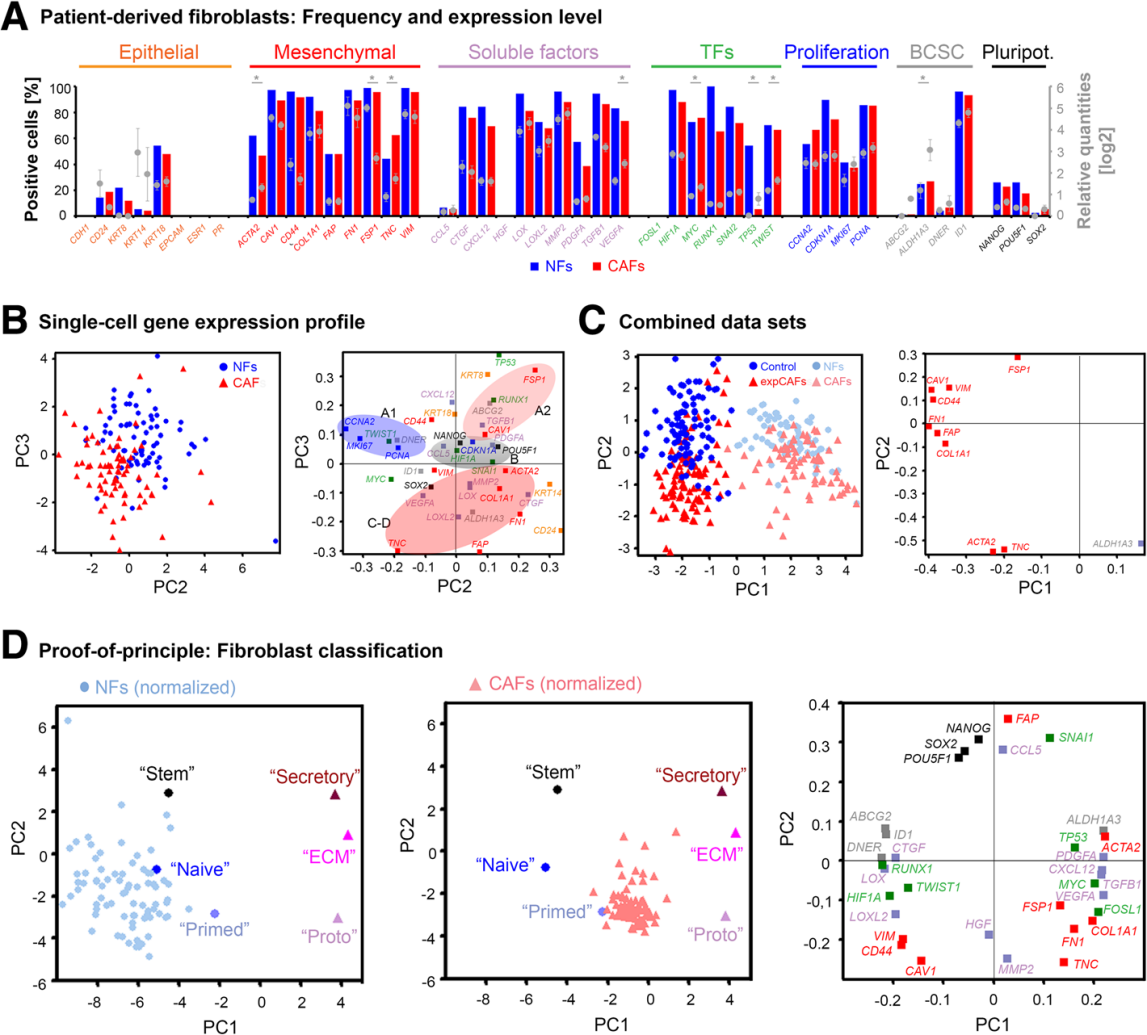
Analysis of patient-derived fibroblasts. **a** Basic statistics of single-cell gene expression profiles of patient-derived fibroblasts isolated from invasive, ERα-positive breast cancer and according normal tissue. Graphs represent frequency of selected gene targets in percentage as bars and average gene expression levels given as log2-transformed relative quantities depicted as dots. Error bars represent SEM (*p-value < 0.05 ANOVA, NF: normal fibroblast, CAF: cancer-associated fibroblast). **b** (*Left panel*) Principal component analysis (PCA) of 152 individual normal (*n* = 77, *blue dots*) and cancer-associated fibroblasts (*n* = 75, *red triangles*). (*Right panel*) Plot represents the gene loadings for the PCA. Principal component projection of the genes illustrates the contribution of each gene to the scores of PC2 and PC3. Groups of genes are indicated as follows; *orange*: epithelial, *red*: fibroblast markers, *purple*: chemokines, *green*: transcription factors, *blue*: proliferation markers, *grey*: breast cancer-specific stem cell markers, *black*: pluripotency. Identified gene clusters of cell line model are highlighted with gene cluster A divided into two (A1, A2) whereas gene cluster E is absent. **c** PCA depicting combined mean-centered datasets of single-cell gene expression of CAF model and patient-derived fibroblasts based on ten genes with potential fibroblast activation-predictive qualities. **d** PCAs of subgroups/differentiation states and applying patient-derived normal (*left panel*) and cancer-associated fibroblast (*middle panel*) single-cell data as test set. Patient data was normalized due to variable global expression levels by autoscaling data per cell. (*Right panel*) According gene loadings for PCAs

With regard to the CAF model, patient-derived normal and according cancer-associated fibroblasts revealed a conserved gene expression, defined by increased transcript levels of *ACTA2*, *COL1A1*, *TNC, VEGFA* and *ALDH1A3,* with concomitant decreased expression of *CAV1, CD44* and *VIM* in CAFs (Fig. 4a).

Likewise, PCA of primary normal fibroblasts and CAFs presents them as distinct molecular cell types (Fig. 4b). The according gene expression pattern was somewhat more convoluted, yet proliferation and pluripotency gene clusters remained. Identified gene clusters of cell line model are highlighted. We observed gene cluster A to be divided into two (A1, A2) marking normal fibroblasts and overlapping gene clusters C-D characterizing fibroblast activation and CAF phenotypes whereas gene cluster E is absent. In order to identify whether and which fibroblast markers contribute the most to a generic CAF phenotype, we combined both data sets, CAF model and primary fibroblasts, by mean-centering data. We found *ACTA2, TNC* and *ALDH1A3* to be the best common CAF markers, however not sufficient for a clear distinction from normal fibroblasts (Fig. 4c). SMAα and Tenascin-C are known fibroblast activation and CAF markers [4], but to the best of our knowledge this is the first report linking an aldehyde dehydrogenase family member to CAF activation.

No clear distinct gene correlations defining normal and activated fibroblasts could be identified for primary cells (Additional file 2B, left panel). Gene cluster A and a minimized gene cluster C/D, attributed to ‘naive’ and ‘proto-myofibroblastic’ states respectively, were identified (Additional file 2B, middle and right panel). Individual gene correlations only hint at a stem cluster exclusive for the normal fibroblasts and a tighter gene co-regulation for CAFs. In line with PCA analysis no gene cluster E was observed, however we further observed an additional correlation cluster including *TNC* and *VEGFA* which was absent for normal fibroblasts (Additional file 2B). It has been suggested that FSP1-positive CAFs expressing Tenascin C and VEGFA represent metastasis-associated fibroblasts [13]. However analysed patient-derived CAFs revealed a pronounced decrease in *FSP1* transcript levels.

Utilizing defined molecular fibroblast differentiation states we applied data of primary cells as test set within a PCA to determine their activation status. We found normal fibroblast to be closest resembling the “naive” phenotype, if somewhat more diverse, ranging from ‘stem-like’ to ‘primed’ than the CAFs which clustered more tightly mainly between the ‘primed’ and ‘protomyofibroblastic’ phenotypes. Thus, generation of a molecular differentiation model allows classification of patient-derived breast cancer-associated fibroblasts as early onset CAFs defined by relatively low *ACTA2* positivity and moderate secretory profile and mostly exhibiting ECM remodelling qualities with high *COL1A, TNC, MMP2, LOX* and *LOXL2* levels with concomitant decrease in *CAV1* and *CD44*.

### Functional analysis of stem potential

Stem cells are unspecialised cells at the apex of a hierarchical cellular organization with the ability to self-renew and give rise to more specialised/ differentiated progeny. The discovery of specific markers and common molecular process underlying the core stem cell properties, also referred to as stemness, has been object of intense study. Stemness potential is typically characterized by high expression of pluripotency genes, tissue-specific stem cell markers and low proliferation rate/quiescence [14].

**Fig. 5:**
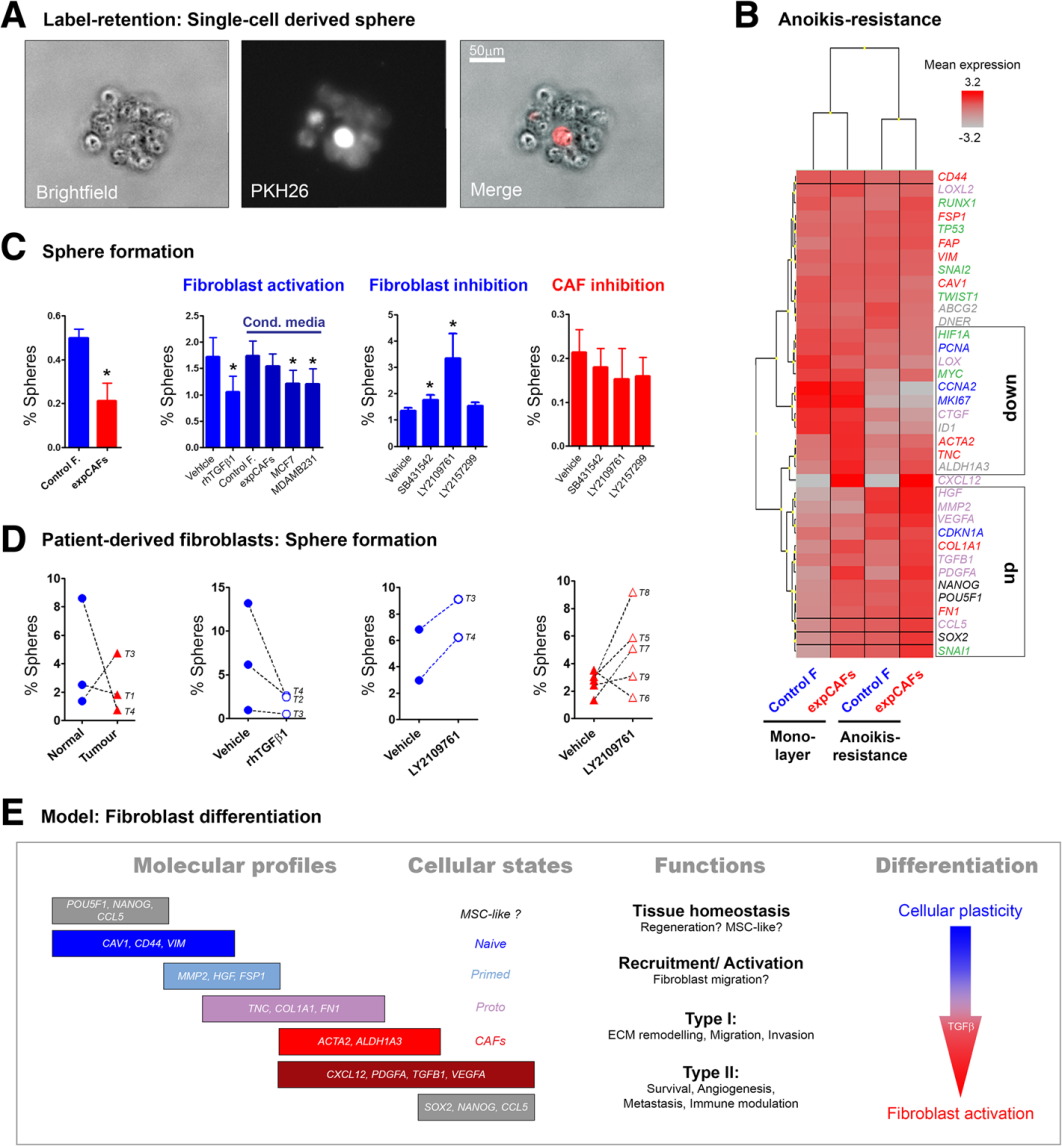
Anoikis-resistance and sphere formation of fibroblast cells. **a** Microscopic picture depicting a sphere derived from a PKH-labelled single cell after 5 days in non-adherent culture conditions. **b** Heatmap (unsupervised clustering) of bulk RNA expression levels before and after 24 h of anoikis resistance. **c** Graphs represent sphere formation capacity as percentage of control (*blue*) and expCAFs (*red*) without and upon treatment with 10 ng/ml of recombinant human TGFβ1 (rhTGFβ1), 72 h cell-conditioned media of breast cancer cell lines (MCF7, MDA-MB-231) or 10uM of TGFβ signalling-targeted pharmacological inhibitors (TGFBR1 inhibitors: SB431542, LY2157299; dual TGFBR1/TGFBR2 inhibitor: LY2109761). Three independent experiments with triplicates were performed. **d** Graphs representing sphere formation as percentage of individual patient-derived fibroblasts (triplicates) depicted as normal-matched (*blue*) and cancer-associated fibroblast (*red*) and paired treatment response. Each sample is marked with corresponding tumour number (T1-T9). Clinical information for each tumour can be found in Additional file 1. **e** Working model of hierarchical fibroblast differentiation comprising diverse activation states with overlapping molecular profiles

Cellular organization of fibroblasts is still obscure owing to diverse cells of origin and modes of activation. In order to validate the existence of a potential hierarchical framework of fibroblast we assessed anoikis resistance/anchorage independence and subsequent sphere formation capacity as functional readout for stem-like characteristics. Label-retention analysis was employed as proof-of-principle confirming the ability of a single cell to give rise to spheroid growth upon survival under non-adherent conditions (Fig. 5a). Furthermore, when we compared RNA expression of conventionally cultured fibroblasts with 24 h anoikis-resistant cells, we noted increased expression of all pluripotency genes and *CCL5* while proliferation genes (*CCNA2, MKI67)* were downregulated (Fig. 5b). In line, *CDKN1A,* encoding p21 which regulates cell cycle arrest/ quiescence was upregulated. Remarkably, fibroblast activation markers *ACTA2, TNC* and *ALDH1A3* showed lower expression whereas ECM markers *COL1A1* and *FN1* and several soluble factors (most prominently *HGF, MMP2*) exhibited higher transcript levels. Taken together, single-cell derived sphere formation and anoikis resistance-mediated increase in pluripotency support validity of our approach to monitor stemness.

Quantifying stem cell-like potential in the fibroblast cell lines we observed significant lower sphere number for expCAFs (Fig. 5c). To test whether paracrine activation of fibroblasts accounts for loss of sphere-forming capacity we transdifferentiated control fibroblast through cultivation with recombinant human TGFβ1 ligand or cancer cell-conditioned media. TGFβ1 and cancer-secreted factors significantly reduced sphere formation whereas the use of pharmacological TGFβ inhibitors increased sphere formation. This potentially highlights a key role of the TGFβ pathway in stem cell maintenance. In contrast inhibiting TGFβ pathway in expCAFs did not affect sphere numbers suggesting an irreversible differentiation state and diminished cellular plasticity of highly activated fibroblasts.

Two out of three patient-derived fibroblasts confirmed reduced sphere-forming capacity in CAFs (Fig. 5d). It is noteworthy that sphere number was considerably higher in primary cells compared to CAF model cell lines. Modulation of TGFβ signalling demonstrated reduced sphere formation in normal fibroblasts using TGFβ ligand and increased sphere number in both, normal fibroblasts and CAFs, upon TGFβ inhibition. Latter observation may be a result of either a retained cellular plasticity of ex vivo fibroblasts, distinct tumour-stromal interaction with the associated tumour or a mixed population of CAFs with discrete anatomic origin. It has been demonstrated that fibroblast isolated from distinct tumour zones exhibit different biomodulatory properties [15]. The recruitment process and functional role of MSCs to the tumour site is still matter of debate [16], however it was shown that about one fifth of CAFs may originate from bone-marrow derived MSCs [17]. Multipotency may thus be distinct from CAFs of other origin. Analysis of more patient samples is required to address this. Overall our data highlight the existence of a subset of cells with stem-like characteristics and suggest a hierarchical cellular organization of fibroblasts.

## Discussion

CAFs are critical components of the tumour stroma and thus present a promising novel treatment option. However, the multifunctional role of CAFs and their fundamental heterogenic nature makes targeting the tumour stroma clinically challenging. Therefore it is essential to characterize underlying molecular processes of tumour-associated fibroblast activation in order to identify relevant markers enabling monitoring and appropriate targeting of potential functionally distinct CAF subtypes.

Within this comprehensive program we demonstrate that normal fibroblasts and their tumour-activated counterparts are molecularly distinct cell types. Beside loss of Caveolin-1 and increased SMAα and Tenascin-C, we report a member of the ALDH family as a novel fibroblast activation marker which may be linked to pro-tumourigenic features such as secretion of angiogenic and chemotactic growth factors (ie. VEGFA, PDGFA, SDF1). We suggest a combination of these markers to determine stromal activation status as no single marker accounts for all CAFs.

We further reveal that CAFs differentiate with simultaneous increase of numerous characteristic fibroblast activation target genes and paracrine factors with a dominating group of genes regulating a specific tumour microenvironmental aspect such as ECM remodelling (‘Type I’) or growth factor/ cytokine secretion (‘Type II’). Based on cell-to-cell similarity and the overlapping gene expression profiles indicating transitional states, we created subgroups in a sequential manner. Generating a pseudo-timeline recapitulating theoretical differentiation stages allowed ultimately for classification of patient-derived fibroblasts to be predominantly of an early-stage phenotype characterized with minimal *ACTA2* induction but ECM-remodelling qualities (‘proto-myofibroblast’) (Fig. 4d).

Notably, an earlier study suggested two distinct CAF subtypes based on either SMAα or FSP1 expression [18]. Both markers have been shown to be protumourigenic. However FSP1 was found to be expressed on resting as well as activated fibroblasts. With regard to our data highlighting FSP1 as a marker for a ‘primed’ cellular state found in normal fibroblasts, we suggest FSP1 as an early marker in fibroblast activation that may be reverted upon SMAα induction. In accordance, we observed *FSP1* profoundly reduced in the patient-derived CAFs compared to the normal counterparts whereas SMAα-encoding *ACTA*2 gene expression level increased together with *TNC* and *ALDH1A3*. Loss of *FSP1* in mice was shown to impair fibroblast motility and reduced metastasis. Strikingly, in line with this, evaluation of clinical aspects of analysed tumour revealed the absence of lymph node metastasis despite the high risk of lymph node involvement for the ERα+/HER2+/Ki67^high^ tumour subtype [19].

In more detail, analysed fibroblasts are derived from a patient with invasive ductal carcinoma molecularly characterized with high Ki67 index, ERα- and PR-positivity and harbouring HER2 amplification (Additional file 1). This expression profile classifies the tumour to be of the luminal B subtype with worse prognosis compared to the ERα-positive luminal A subtype [20]. Further, the histopathologically observed vascular invasion is associated with unfavourable outcome [21]. However, the tumour presented with residual DCIS which was shown to be associated with favourable outcome [22] and most importantly the tumour displayed no lymph node involvement which is most significant prognostic indicator for patients with early-stage breast cancer [21].

Taking together the tumours clinico-pathological parameters and CAFs’ gene expression profiling it is tempting to assume that despite aggressive features such as big tumour size, high Ki67 status and vascular invasion, that the presence of DCIS, absence of lymph node metastasis and lack of chemotactic “secretory type II CAFs” marks the onset of a spreading tumour which has not yet manifested. With this in mind, analysing CAF-specific markers could be of great prognostic and/or predictive value aiding patient selection and thus advancing personalised medicine.

Our study further highlights the existence of stem-like cells as a physiological feature of tissue-resident fibroblasts. Putative stem-like cells were defined as expressors of pluripotency genes and surviving non-adherent conditions which induces or enriches for pluripotency gene expression, and give rise to single-cell derived spheres. Strikingly, pluripotency correlated with *CCL5* expression, which is a prominent factor in stromal gene expression signatures and linked to worse prognosis in breast cancer [12, 23]. It has previously been demonstrated that fibroblastic cells within the tumour stroma possess mesenchymal stem cell (MSC) qualities and that breast cancer cells stimulate secretion of CCL5, which in turn facilitates its metastatic capacity [12]. Fibroblasts are likely to be more restricted in their differentiation potential than multipotent MSCs, but they may retain some cellular plasticity which appears to be regulated by TGFβ. Very few studies have reported an anoikis-resistant phenotype of fibroblasts, however with a protective role of TGFβ on survival of differentiated fibroblasts [24, 25] which may be a distinct functional feature compared to assessing single-cell survival with subsequent sphere formation. Notably, CAFs appear to be associated with a loss in sphere-forming capacity potentially representing a more differentiated cell type with diminished regenerative ability or cellular plasticity. However it remains to be determined how stem-like CAFs differ functionally from normal stem-like fibroblasts and whether their regulation is dependent on the associated tumour type or other microenvironmental factors.

Of note, a recent review by Kalluri suggests that resting or quiescent fibroblasts may be considered adult tissue-resident mesenchymal stem cell [26]. Even further our data presented here is in line with the hypothesized three main fibroblast phenotypes: (1) resting or quiescent fibroblasts with MSC capacity, (2) normal or wound-healing-associated activated fibroblasts (NAFs) with increased ECM synthesis, secretion and motility thus representative of “primed” fibroblasts and (3) CAFs which exert an enhanced secretory profile while concomitantly associated with loss of contractility (SMAα) and decreased synthetic activity (collagen, fibronectin) compared to NAFs. However transitional stages or cellular states need to be further defined.

## Conclusion

Collectively our data indicate that underlying the cellular heterogeneity, fibroblasts may be hierarchically organized (see model Fig. 5e). The notion of cancer as “a wound that never heals” implies parallels of the transcriptional program between fibroblasts in the tumour stroma and during wound response. We propose that tumour-secreted factors such as TGFβ recruit and activate resident ‘naive’ fibroblasts. A subsequent increase in ECM deposition and ECM-remodelling enzymes is ‘priming’ fibroblasts to a more differentiated state whereas loss of particular mesenchymal markers such as Caveolin-1 may indicate a less reversible commitment of fibroblast activation. This ultimately results in archetypical CAFs with excessive matrix production (‘Type I’) and secretion of angiogenic and metastatic growth factors and immunomodulatory cytokines (‘Type II’). During the fibroblast differentiation, physiological stem or regenerative potential may be lost, mediated by tumour-secreted factors such as TGFβ which in turn effectively induces fibroblast activation.

Distinct fibroblast specialization may depend on cell of origin [27] and it has been noted that heterogeneity and interactions of different types of CAFs potentiates tumour-promoting qualities [28]. Findings reported herein relate to the ERα-positive breast cancer subtype and tissue-resident derived fibroblasts. It has been noted that the majority of CAFs are derived from local stem or progenitor cells whereas about a third may have different cellular origins [28]. We present evidence that tissue-derived fibroblastic cells provide a local source for CAFs and contribute to the immunomodulatory function of the reactive tumour stroma. Overlapping marker expression pattern may indicate transitional cellular states rather than distinct cell types and predominant cellular states of CAFs may be inherent or affected by extrinsic factors such as genetic makeup of the tumour or tumour stage.

Further characterization of individual tumour stroma phenotypes will expand opportunities to discover novel clinical biomarkers and therapeutic targets. Categorizing patient groups with respect to CAFs in combination with current diagnostic tests will enable a more reliable selection of therapeutic strategies that block the tumour microenvironment.

## Additional files

Additional file 1: Characterization of patient-derived fibroblasts and clinical information of used tumours. Fibroblast morphology and SMAα expression levels of isolated normal and cancer-associated fibroblasts. Clinical information of all analysed tumours. (PDF 1033 kb)

Additional file 2: Gene correlation analysis. Heatmaps depicting gene correlation analyses according to fibroblast subgroups (normal versus cancer-activated) for CAF cell line model and primary fibroblasts. (PDF 2716 kb)

## Abbreviations

7AAD: 7-amino-actinomycin D
ABCG2: Adenosine triphosphate-binding cassette sub-family G member 2
ACTA2: Actin, alpha 2, smooth muscle, aorta
ALDH1A3: Aldehyde dehydrogenase 1 family member A3
ANOVA: Analysis of variance
BCSC: Breast cancer-specific stem cells
BSA: Bovine serum albumin
CAFs: Cancer-associated fibroblasts
CAV1: Caveolin 1
CCL5: C-C motif chemokine ligand 5
CCNA2: Cyclin A2
CD24: Cluster of differentiation 24
CD44: Cluster of differentiation 44
CDH1: Cadherin 1
CDKN1A: Cyclin dependent kinase inhibitor 1A
cDNA: Complementary deoxyribonucleic acid
CO_2_: Carbon dioxide
COL1A1: Collagen, type I, alpha 1
CTGF: Connective tissue growth factor
CXCL12: C-X-C motif chemokine ligand 12
DNA: Deoxyribonucleic acid
DNase: Deoxyribonuclease
DNER: Delta/notch like epidermal growth factor repeat containing
ECM: Extracellular matrix
EPCAM: Epithelial cell adhesion molecule
ERα: Oestrogen receptor alpha
ESR1: Oestrogen receptor 1
expCAFs: Experimentally generated cancer-associated fibroblasts
FACS: Fluorescence-activated cell sorter
FAP1: Fas-associated phosphatase 1
FBS: Foetal bovine serum
FN1: Fibronectin 1
FOSL1: Fos-like antigen 1
FSP1: Fibroblast-specific protein 1
GAPDH: Glyceraldehyde-3-phosphate dehydrogenase
HGF: Hepatocyte growth factor
HIF1A: Hypoxia-induced factor 1 alpha
ID1: Inhibitor of deoxyribonucleic acid binding 1
KRT14: Keratin 14
KRT18: Keratin 18
KRT4: Keratin 4
LOX: Lysyl oxidase
LOXL2: Lysyl oxidase like 2
MKI67: Marker of proliferation Ki-67
MMP2: Matrix metallopeptidase 2
MSC: Mesenchymal stem cell
NANOG: Nanog homeobox
PCA: Principle component analysis
PCNA: Proliferating cell nuclear antigen
PCR: Polymerase chain reaction
PDGFA: Platelet-derived growth factor
PGR: Progesterone receptor
poly-HEMA: Poly-2-hydroxyethyl methacrylate
POU5F1: POU class 5 homeobox 1
RNA: Ribonucleic acid
RNase: Ribonuclease
RT-PCR: Reverse transcriptase polymerase chain reaction
RUNX1: Runt-related transcription factor 1
SEM: Standard error of mean
SMAα: Smooth muscle actin alpha
SNAI1: Snail family transcriptional repressor 1
SNAI2: Snail family transcriptional repressor 2
SOM: Kohonen self-organizing maps
SOX2: Sex determining region Y-box 2
TGFB1: Transforming growth factor beta 1
TGFβ: Transforming growth factor beta
TNC: Tenascin C
TP53: Tumor protein p53
TWIST1: Twist family basic helix-loop-helix transcription factor 1
VEGFA: Vascular endothelial growth factor A
VIM: Vimentin
