# Supplementary material for: Cellular organization and molecular differentiation model of breast cancer-associated fibroblasts
Source: Mol Cancer. 2017 Apr 3;16:73. doi: 10.1186/s12943-017-0642-7 (PMC5376683; doi:10.1186/s12943-017-0642-7)
Supplement: Supplementary file 1 — Characterization of patient-derived fibroblasts and clinical information of used tumours. Fibroblast morphology and SMAα expression levels of isolated normal and cancer-associated fibroblasts. Clinical information of all analysed tumours. (PDF 1033 kb) [file 12943_2017_642_MOESM1_ESM.pdf]

Additional file 1

(A) Patient-derived fibroblasts used for single-cell gene expression profiling (T1)

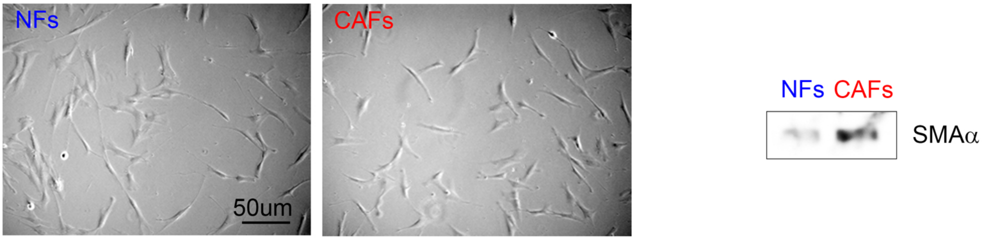

Clinical data:

Invasive ductal breast carcinoma with associated grade 3 DCIS

Age	42 years
Tumour size	88 mm
Vascular invasion	yes
Lymph node involvement	no

ERα	90%
PR	30%
Ki67	70%
HER2	70%, amplified (SISH)

(B) Clinical information of all used tumours (T1-T9)

Tumour	Age (years)	Type	Size (mm)	VI	LN	TILs	ERα	PR	Ki67	HER2	Molecular subtype
T1	42	ductal (DCIS)	88	yes	0	na	90%	30%	70%	2+ (ampl)	Luminal B (Her2+)
T2	79	lobular	79	no	0	10-49%	80%	30-50%	31%	3+ (ampl)	Luminal B (Her2+)
T3	62	ductal	33	no	0	1-9%	100%	80%	21%	1+	Luminal A
T4	56	ductal	34	no	0	1-9%	90%	80%	14%	1+	Luminal A
T5	94	lobular	38	no	0	1-9%	100%	100%	<15%	0	Luminal A
T6	45	ductal	41	no	0	>75%	0	0	80%	2+	Her2+
T7	81	ductal	39	yes	0	50-74%	0	0	80%	2+ (ampl)	Her2+
T8	89	ductal (DCIS)	75	no	0	10-49%	0	0	80%	0	Basal
T9	83	ductal	59	yes	28/32	10-49%	100%	90%	55%	2+ (ampl)	Luminal B (Her2+)

Additional file 1. Characterization of patient-derived fibroblasts. (A) Microscopic image of isolated fibroblast cells (upper left) and western blot of smooth muscle actin-alpha (SMAα) (upper right). Clinico-pathological parameters and prognostic molecular markers of tumour used for fibroblast iolation and subsequent single-cell gene expression profiling. (Lower panel) (B) Clinical information of all used tumours. (NFs: normal fibroblasts, CAFs: cancer-associated fibroblasts, DCIS: ductal carcinoma *in situ*, ERα: oestrogen receptor, PR: progesterone receptor, HER2: human epidermal growth factor receptor 2, SISH: silver *in situ* hybridization, VI: vascular invasion, LN: lymph node metastasis, TILs: tumour infiltrating lymphocytes, na: data not available, ampl: amplified, HER2 scoring: 0 negative, 1+ also negative, 2+ borderline, 3+ positive)
