# Supplementary material for: Cellular organization and molecular differentiation model of breast cancer-associated fibroblasts
Source: Mol Cancer. 2017 Apr 3;16:73. doi: 10.1186/s12943-017-0642-7 (PMC5376683; doi:10.1186/s12943-017-0642-7)
Supplement: Supplementary file 2 — Gene correlation analysis. Heatmaps depicting gene correlation analyses according to fibroblast subgroups (normal versus cancer-activated) for CAF cell line model and primary fibroblasts. (PDF 2716 kb) [file 12943_2017_642_MOESM2_ESM.pdf]

Additional file 2

Gene correlations

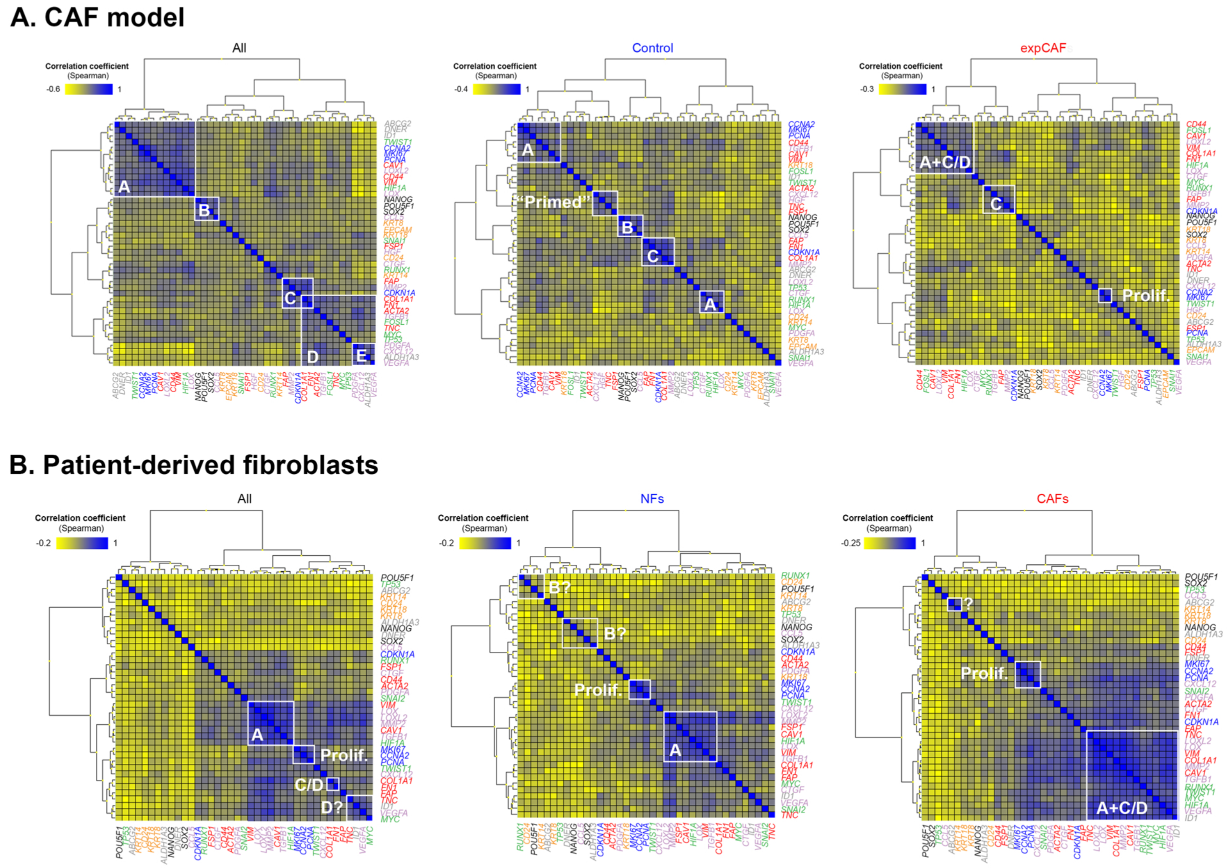

Additional file 2. Gene correlation analysis. (A) Heatmap (unsupervised clustering, Euclidian distance) demonstrating gene correlations coefficients (Spearman) based on gene expression of all cells (left panel), control only (middle) and experimentally-generated cancer-associated fibroblasts (expCAFs) only (right panel). (B) Heatmap (unsupervised clustering, Euclidian distance) demonstrating gene correlations coefficients (Spearman) based on gene expression of primary fibroblasts (left panel), normal fibroblasts only (middle) and CAFs only (right panel). Gene clusters A-E are according to Figure 2A. Additional clusters are noted.
